# Insulation Foam Concrete Nanomodified with Microsilica and Reinforced with Polypropylene Fiber for the Improvement of Characteristics

**DOI:** 10.3390/polym14204401

**Published:** 2022-10-18

**Authors:** Besarion Meskhi, Alexey N. Beskopylny, Sergey A. Stel’makh, Evgenii M. Shcherban’, Levon R. Mailyan, Nikita Beskopylny, Andrei Chernil’nik, Diana El’shaeva

**Affiliations:** 1 Department of Life Safety and Environmental Protection, Faculty of Life Safety and Environmental Engineering, Don State Technical University, Gagarin Sq. 1, 344003 Rostov-on-Don, Russia; 2 Department of Transport Systems, Faculty of Roads and Transport Systems, Don State Technical University, Gagarin Sq. 1, 344003 Rostov-on-Don, Russia; 3 Department of Unique Buildings and Constructions Engineering, Don State Technical University, Gagarin Sq. 1, 344003 Rostov-on-Don, Russia; 4 Department of Engineering Geology, Bases, and Foundations, Don State Technical University, 344003 Rostov-on-Don, Russia; 5 Department of Roads, Don State Technical University, 344003 Rostov-on-Don, Russia; 6 Department of Hardware and Software Engineering, Don State Technical University, Gagarin Sq. 1, 344003 Rostov-on-Don, Russia

**Keywords:** polymer fiber, foam concrete, polypropylene fiber, silica fume, nanomodification

## Abstract

Some of the primary problems of construction are brittleness and low the mechanical properties of good thermal insulation materials. Heat-insulating foam concrete has a low thermal conductivity. However, it is practically impossible to transport it over long distances since corners are cracked during transportation, the structure is broken, and, in principle, the fragility of this material is a big problem for modern buildings. The purpose of this study was to develop a heat-insulating foam concrete with improved characteristics by experimentally selecting the optimal dosage of polypropylene fiber and a nanomodifying microsilica additive. Standard methods for determining the characteristics of fiber foam concrete were used as well as the method of optical microscopy to study the structure of the composite. It has been established that the use of polypropylene fiber with the optimal reinforcement range from 1% to 3% allows us to achieve an improvement in the mechanical and physical characteristics of fiber foam concrete. The optimal dosage of the nanomodifier introduced instead of a part of the binder (10%) and polypropylene fiber (2%) by weight of the binder was determined. The maximum values of increments in mechanical characteristics were 44% for compressive strength and 73% for tensile strength in bending. The values of the thermal conductivity coefficient at optimal dosages of the nanomodifier and fiber decreased by 9%. The absence of microcracking at the phase boundary between the polypropylene fiber and the hardened cement–sand matrix due to nanomodification was noted.

## 1. Introduction

Some of the main problems of construction are fragility and the low mechanical performance of good thermal insulation materials. So, low-density foam concrete has good thermal insulation ability, but it is practically impossible to transport it over long distances since corners are broken during transportation, the structure is broken, and, in principle, the fragility of this material is a big problem for modern builders. In this regard, the actual direction of building materials science is the search for ways to find the optimal relationship between the density and strength of heat-insulating concretes. At the same time, modern materials science puts forward the creation of new and improvement of existing building materials and technologies for their production as the main tasks.

The most relevant and progressive building materials include heat-insulating concretes, among which foam and fiber foam concretes stand out—some of the most popular in practical construction due to their properties, economy, reliability, and durability [1,2]. Foam concrete is one of the types of cellular concrete, and this type of concrete belongs to lightweight concrete, and its density can vary from 300 to 1200 kg/m^3^. The structure of this material is characterized by a multitude of randomly arranged air voids, which were formed in the concrete mixture due to the addition of a foaming agent [3,4]. As for the advantages of this material, several researchers [5,6,7] noted that foam concrete has properties such as low density and thermal conductivity, this material is also fire resistant, and, due to its porous structure, absorbs sound well. Table 1 compares foam concrete and aerated concrete in terms of main raw materials, production method, and environmental impact.

The main raw materials for the preparation of foam concrete are cement, foaming agent, sand, and water for mortar. Ordinary Portland cement, quick-hardening Portland cement, sulfate-resistant Portland cement, and high alumina cement can be used as a binder in foam concrete and as additional materials to replace part of the cement in the range from 10% to 70% in place of materials such as microsilica, fly ash, lime, ground granulated blast-furnace slag, and others. As a rule, various additional components in addition to the main raw material are used to improve the mechanical and physical characteristics [14,15,16,17,18,19,20,21,22,23,24,25]. Table 2 provides an overview of various ways to modify and improve the physical–mechanical characteristics by introducing additional components.

After considering the modifiers and nanomodifiers that are used for concrete, another important criterion for the compatibility of these nanomodifiers with foam concentrates should be noted. The blowing agent is a key component for foam concrete and regulates the density of concrete due to the amount of air bubbles formed in the cement paste mixture. Protein-based and synthetic-based foam concentrates are the most common in practice; protein-based foam concentrates are characterized by the formation of a more durable and closed-cell structure with a stable network of air cavities. When using synthetic foam concentrates, this causes much greater expansion and makes it possible to achieve a lower density [34,35,36,37,38]. In addition, the use of nanomodifiers of various types and dispersion together with fiber further improves the quality of concrete and contributes to obtaining improved composites both in terms of structure and in terms of mechanical and physical characteristics [33,39,40,41,42].

The goal of this study is to develop a heat-insulating foam concrete with improved characteristics by experimentally selecting the optimal dosage of polypropylene fiber and a nanomodifying microsilica additive. Tasks to achieve the set goal are as follows:

(1) Determination of a rational optimal dosage of polypropylene fiber, which allows to achieve the maximum improvement in the mechanical and physical characteristics of fiber foam concrete;
(2) Determination of the optimal dosage of the nanomodifying microsilica additive;
(3) Revealing the dependences of the mechanical and physical characteristics of fiber foam concrete on the values of dosages of polypropylene fiber and silica fume;
(4) Determination of further research prospects.

The scientific novelty of the work lies in obtaining new dependences of the characteristics of foam concrete on the dosages of polypropylene fiber and a nanomodifying microsilica additive. Theoretical and experimental studies were carried out to evaluate the effectiveness of the use of polypropylene fibers in foam concrete technology, both separately and in combination with a nanomodifying additive. According to the results of analytical studies, the optimal ranges of the percentage of fiber reinforcement were previously determined and, during experimental studies, the most optimal dosages of fiber and silica fume were determined, at the values of which the best physical and mechanical characteristics of foam concrete are observed.

## 2. Materials and Methods

### 2.1. Materials

To ensure the correctness and reliability of the results obtained, a methodological decision was made to use Portland cement without additives, which made it possible to evaluate the influence of additives and their complex effect, regardless of the additives contained in the Portland cement. As the initial filler, quartz sand with known characteristics was taken because quartz sand is not reactive, and it allows us to evaluate the influence of certain modifiers in its pure form so that the scientific novelty is distinguished by reliability, correctness, reproducibility, and repeatability. Foaming agent PB-2000 was adopted based on the results of a literature review [34,35,36,37,38]. The blowing agent used is well studied. When tested empirically by various authors of its compatibility with other additives of both mineral and organic origin, no undesirable, non-recommended consequences were noticed; therefore, this particular foaming agent PB-2000 was adopted. Polypropylene fiber was taken in accordance with Table 3 produced by Fibropolymer, since all its characteristics are established and verified. The specified fiber has proven itself well in the cellular concrete market in the Eurasian region. The characteristics of the main raw materials used in this study are presented in Table 3.

The properties of Portland cement were tested according to GOST 30744 «Cements. Methods of testing with using polyfraction standard sand», sand, according to GOST 8735 «Sand for construction work. Testing methods». The properties of the foaming agent and fiber are taken from the manufacturer.

**Figure 1:**
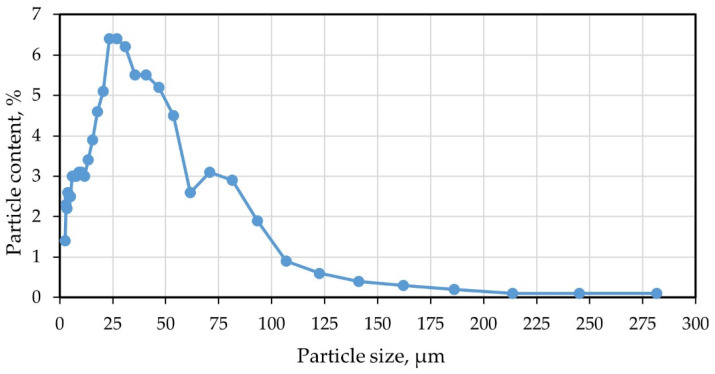
Granulometric composition of microsilica.

Microsilica MK-85 (Novolipetsk Iron and Steel Works, Lipetsk, Russia) was used as a modifying additive for scientific research. The chemical composition of microsilica is presented in Table 4, and Figure 1 shows its granulometric composition (performed on a laser analyzer Microsizer 201C (VA Instalt, St. Petersburg, Russia) [40]).

It follows from Figure 1 that the largest number of microsilica particles (more than 65%) are in the size range from 8 µm to 60 µm. The granulometric composition was determined based on the results of our own research and verified according to the data of the manufacturing plant. A good correlation of the data obtained makes it possible to take these characteristics as design characteristics when designing the compositions of nanomodified fiber foam concretes and makes it possible to regulate the structure and control the properties of the resulting new type concretes.

Thus, all received materials, after their additional verification and comparison of their own laboratory data with the data of manufacturing plants, made it possible to set and determine specific research methods.

### 2.2. Methods

Research methods were based on normative and technical proven methods and were reduced to two stages:

(1) Making samples;
(2) Testing and examination of the obtained samples.

Foam concrete samples were made in laboratory conditions. For the manufacture of all samples, the following parameters of concrete mixtures were adopted [43]:

- Water-solid ratio W/T = 0.49;
- The ratio of sand and cement P/C = 0.3;
- Foaming agent content PO = 5.25 kg/m^3^.

The sand was preliminarily sifted through a standard set of KSI sieves (Vibrotechnik, St. Petersburg, Russia) to obtain the required fraction of 0.16–0.315 mm.

The dosed ingredients—water, Portland cement, microsilica, fiber, and sand—were successively loaded into a CA 400/500 laboratory turbulent mixer with a capacity of 50 L and a rotor speed of 620 rpm (Don State Technical University, Rostov-on-Don, Russia). Mixing of all components was carried out until the fiber was evenly distributed over the volume of the concrete mixture. Next, with the mixer activator stopped, a dosed foaming agent was poured in and the mixture was poured for 4 min. The prepared mixtures were poured into metal molds previously lubricated with mineral oil. Forms with foam and fiber-reinforced concrete mixtures were slightly compacted by tapping on a metal table and smoothing with a ruler. Further, the samples hardened under normal conditions with a temperature of (20 ± 2) °C and relative humidity of the air (95 ± 5)%. After 3 days after manufacture, the samples were removed from the molds, and they continued to harden under normal conditions until reaching 28 days of age. The sequence, time, speed of mixing, and the order of introduction of materials are adopted according to previous studies [43]. Testing of prototypes was carried out using the following test equipment and measuring instruments:

- Hydraulic press IP-1000 (NPK “TEKHMASH”, Neftekamsk, Russia);
- Device for determining thermal conductivity ITP-MG4 (SKB Stroypribor, Chelyabinsk, Russia);
- Laboratory oven ShS-80-01 SPU (Smolenskoe SKTB SPU, Smolensk, Russia);
- Metal measuring ruler 500 mm (Stavropol Tool Plant, Stavropol, Russia);
- Laboratory scales HT-5000 (NPP “Gosmetr”, St. Petersburg, Russia); caliper ShTs-I-250–0.05 (NPP “Chelyabinsk Tool Plant”, Chelyabinsk, Russia);
- A device for measuring deviations from the plane NPL-1 and a device for measuring deviations from perpendicularity NPR-1 (RNPO RusPribor, St. Petersburg, Russia).

The study of the structure of fiber foam concrete samples was carried out on an MBS-10 microscope (Izmeritelnaya Tekhnika, Moscow, Russia).

The physical and mechanical characteristics of the fiber foam concrete samples and the control composition were determined by the following methods:

- Compressive and tensile strength in bending was determined according to GOST 10180 “Concretes. Methods for strength determination using reference specimens”;
- The average density was determined according to GOST 12730.1 “Concretes. Methods of determination of density”;
- Release humidity was determined according to GOST 12730.2 “Concretes. Method of determination of moisture content”;
- Thermal conductivity was determined according to GOST 7076 “Building materials and products. Method of determination of steady-state thermal conductivity and thermal resistance”.

**Figure 2:**
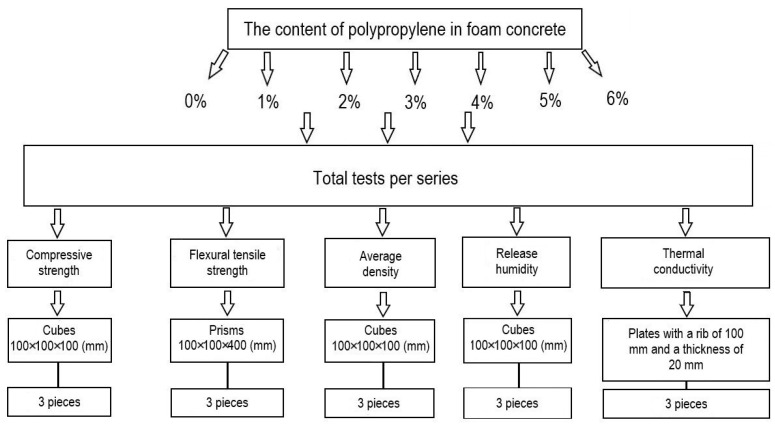
Design of the preliminary study.

Before conducting the main part of the experimental studies to study the effect of the content of microsilica additive introduced instead of a part of the cement and the percentage of dispersed reinforcement with polypropylene fiber on the physical and mechanical characteristics of fiber foam concrete, preliminary studies were carried out to identify the optimal range of the percentage of fiber reinforcement, the plan of which is shown in Figure 2.

During the main part of the experimental studies, a total of 22 series of samples were made and tested with different values of the percentage of fiber reinforcement with polypropylene fiber and the content of the nanomodifying microsilica additive. The experimental research program is shown in Table 5.

## 3. Results and Discussion

### 3.1. Physical and Mechanical Characteristics of Fiber Foam Concrete Samples without Nanomodifying Additive

**Figure 3:**
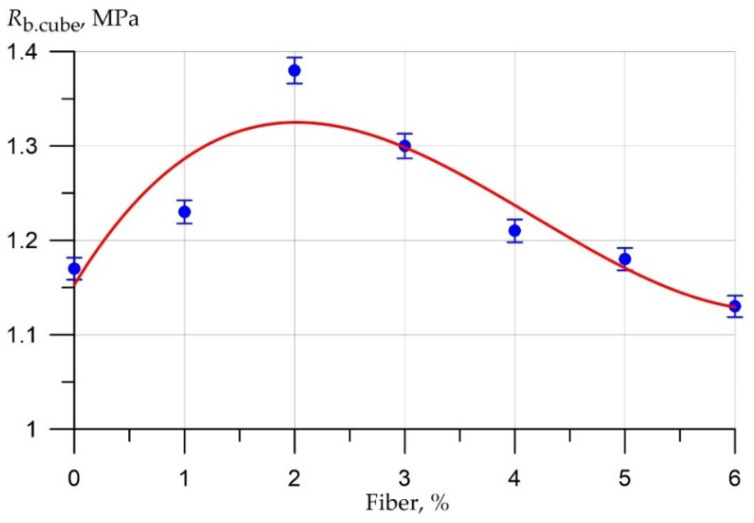
The results of experimental studies of the compressive strength (R_b.cube_) of prototypes of fiber foam concrete at various dosages of polypropylene fiber.

**Figure 4:**
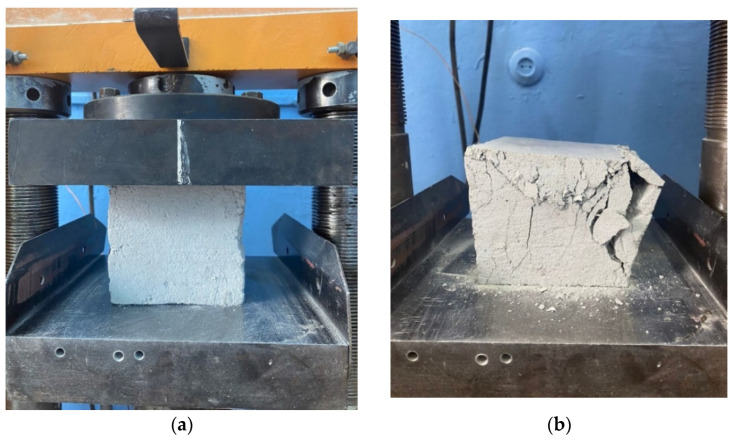
Sample of fiber-reinforced concrete under compression test (graphic scale is 1:3): (**a**) before loading and (**b**) after loading.

**Figure 5:**
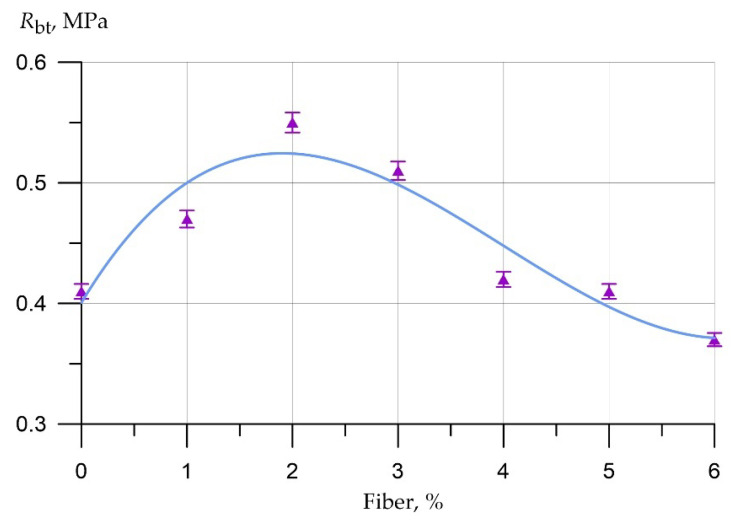
Results of experimental studies of tensile strength in bending (R_bt_) of fiber foam concrete samples at various dosages of polypropylene fiber.

**Figure 6:**
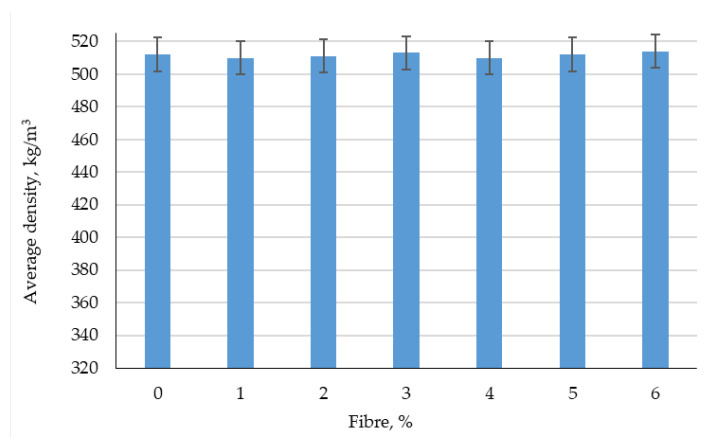
The results of experimental studies of the average density of prototypes of fiber foam concrete at various dosages of polypropylene fiber.

**Figure 7:**
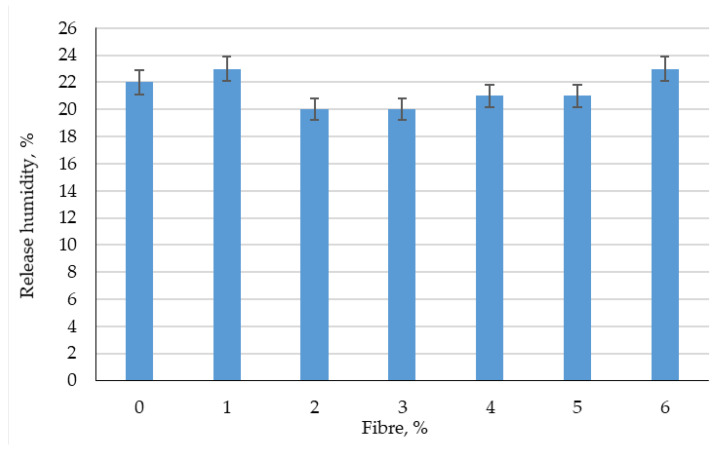
Results of experimental studies of the release moisture content of fiber foam concrete samples at various dosages of polypropylene fiber.

**Figure 8:**
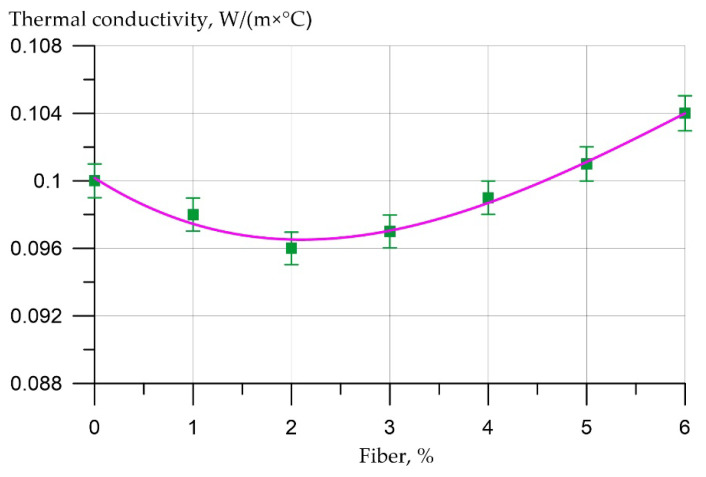
Results of experimental studies of the thermal conductivity of fiber foam concrete samples at various dosages of polypropylene fiber.

The results of experimental studies of the effect of the percentage of fiber reinforcement on the physical and mechanical characteristics of foam concrete are shown in Figure 3, Figure 4, Figure 5, Figure 6, Figure 7 and Figure 8.

The compressive strength curve of the test samples of fiber foam concrete (Figure 3) at various dosages of polypropylene fiber is approximated by a polynomial of the third degree:

(1) $$R_{b.\mathrm{cub}}\;=\;1.153\;+\;0.191\;x\;-\;0.0625x^{2}\;+\;0.005x^{3},\;\;\;\;\;\;R^{2}\;=\;0.833$$

Here *x* is the dosage of polypropylene fiber; *R*^2^ is the coefficient of determination.

Figure 3 shows that the best range of fiber reinforcement is the fiber content from 1% to 3%, and in this range there is an increase in compressive strength compared to the control sample. Thus, the increase in compressive strength was 5% at a dosage of 1% fiber, and at dosages of 2% and 3%, the increases were 18% and 11%, respectively. A rather sharp drop in compressive strength is observed when the content of polypropylene fiber is 4% or more. So, the increase in strength at a fiber dosage of 4% and 5% was only 3% and 1%, respectively, and at a dosage of 6%, a drop in compressive strength is observed compared to the control composition, up to 3%.

Figure 4 shows photographs of the process of testing fiber concrete in terms of “compressive strength”.

Figure 4b shows the viscous nature of the destruction of a sample of fiber foam concrete with polypropylene fiber. The fiber restrains the destruction of the foam concrete sample under load and eliminates brittle fracture. Samples do not crumble.

The dependence curve of tensile strength in bending of fiber foam concrete samples at various dosages of polypropylene fiber is approximated by the equation:

(2) $$R_{\mathrm{bt}}\;=\;0.40\;+\;0.145x\;-\;0.05x^{2}\;+\;0.00416x^{3},\;\;\;\;\;\;R^{2}\;=\;0.89$$

The change in tensile strength in bending is similar to the nature of the change in compressive strength. Thus, the increases in tensile strength in bending at dosages of polypropylene fibers of 1%, 2%, 3%, 4%, and 5% were 15%, 34%, 24%, 2%, and 0%, respectively. At a fiber dosage of 6%, the drop in strength was 10%.

Control and evaluation of the strength of fiber foam concrete samples were carried out in accordance with GOST 18105 “Concretes. Rules for control and assessment of strength”. The standard deviation of strength values does not exceed 5%, and the coefficient of strength variation is not more than 4%, which meets the requirements of the standard.

From Figure 6 and Figure 7, it follows that dispersed reinforcement with polypropylene fiber in an amount of 1–6% by weight of the binder does not significantly affect the change in average density and release moisture.

The control and evaluation of the average density of fiber foam concrete samples was carried out in accordance with GOST 27005 “Light-weight and cellular concretes. Rules for control and assessment density” and for release moisture in accordance with GOST 13015 “Concrete and reinforced concrete products for construction. General technical requirements. Rules for acceptance, marking, transportation and storage”. The variation in the results of the average density (no more than 2%) and release moisture (no more than 4.5%) of each sample turned out to be less than the ranges allowed by the standards.

The dependence of the thermal conductivity of fiber foam concrete samples at various dosages of polypropylene fiber is approximated by the equation

(3) $$T_{c}\;=\;0.100\;-\;0.00371x\;+\;0.00106x^{2}\;-\;5.55\times 10^{-5}\;x^{3},\;\;\;\;\;\;\;R^{2}\;=\;0.98$$

From Figure 8, it follows that the use of polypropylene fiber had little effect on the change in the thermal conductivity of fiber foam concrete. Thus, at a dosage of fiber in the amount of 1%, the thermal conductivity value decreased to 2%, and at dosages of 2%, 3%, 4%, and 5%, the thermal conductivity of fiber foam concrete decreased by 4%, 3%, 1%, and increased by 1%, respectively, and at a dosage 6% it increased by 4%.

The control and evaluation of the thermal conductivity of fiber foam concrete samples was carried out in accordance with GOST 7076 “Building materials and products. Method of determination of steady-state thermal conductivity and thermal resistance”. The variation of thermal conductivity results for each sample does not exceed 2% and turned out to be less than the ranges allowed by the standards.

According to the results of a study aimed at identifying the optimal range of fiber reinforcement, it was found that the most optimal range of polypropylene fiber is its content in foam concrete in an amount of 1% to 3%. The best values of the physico-mechanical characteristics of fiber foam concrete were recorded when the fiber dosage was 2% by weight of cement. Later, when conducting experimental studies on the production of fiber foam concrete nanomodified with the addition of microsilica, the range of dispersed reinforcement in the amount of 1–3% was adopted.

### 3.2. Mechanical Characteristics of Prototypes of Fiber Foam Concrete Modified with the Addition of Microsilica

**Figure 9:**
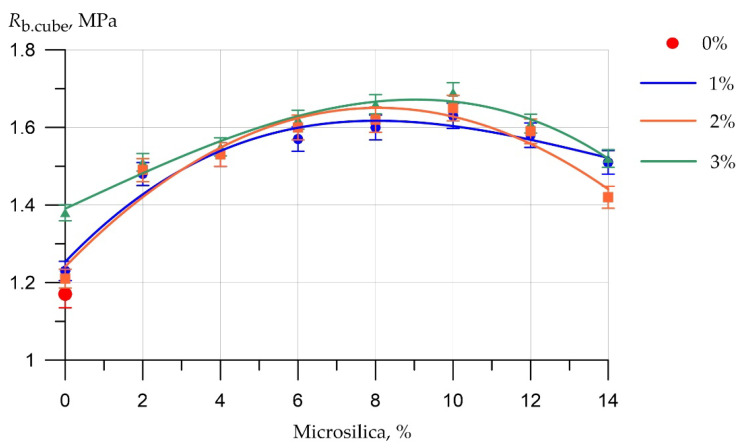
The results of determining the compressive strength of fiber foam concrete samples at various dosages of microsilica introduced instead of a part of the binder and dosages of polypropylene fiber of 0%, 1%, 2%, 3%.

**Figure 10:**
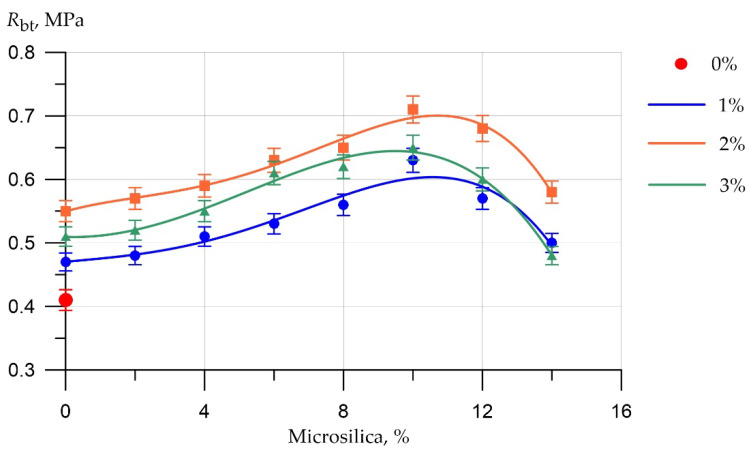
Results of experimental studies of tensile strength in bending of fiber foam concrete samples at various dosages of microsilica introduced instead of a part of the binder and dosages of polypropylene fiber of 0%, 1%, 2%, 3%.

The results of experimental studies of the influence of the content of microsilica additive introduced instead of part of the cement and the percentage of dispersed reinforcement with polypropylene fiber on the strength characteristics of fiber foam concrete are presented in Figure 9 and Figure 10.

The graph in Figure 9 shows the dependence of the compressive strength of fiber foam concrete samples on the percentage of replacement of the part of the cement with silica fume and on the percentage of fiber reinforcement.

Based on the values of the compressive strength of the fiber foam concrete samples, it can be said that the most optimal dosage of silica fume, introduced instead of a part of the cement, is a dosage of 10%, since it is at this percentage of replacement that the highest compressive strength value was recorded, which amounted to 1.69 MPa for the composition of the type F14. As for the most optimal dosage of polypropylene fiber, its value is 2% by weight of the binder component. Compositions of type F2, F5, F8, F11, F14, F17, and F20 at different dosages of microsilica, but the same value of dispersed reinforcement equal to 2%, showed the best values of compressive strength, which are respectively equal to 1.51 MPa, 1.55 MPa, 1.62 MPa, 1.63 MPa, 1.69 MPa, 1.61 MPa, and 1.52 MPa.

The graph in Figure 10 shows the dependence of the tensile strength in the bending of fiber foam concrete samples on the percentage of replacing part of the cement with silica fume and on the percentage of fiber reinforcement.

The flexural tensile data results shown in Figure 10 have a similar pattern to the results obtained from the compressive strength tests. Accordingly, the values of the optimal dosages of silica fume and fiber are similar to those for compressive strength. Higher increases in tensile strength in bending are a consequence of the work of the fibers. Polypropylene fibers introduced into the foam concrete mixture after concrete hardening are reliably connected to it due to adhesion forces and key engagement along the surface of the curvilinear fiber at the ends of the fiber. Due to this key engagement, concrete and fiber work together much better, resisting the effects of tensile stresses.

### 3.3. Physical Characteristics of Prototypes of Fiber Foam Concrete Modified with the Addition of Microsilica

**Figure 11:**
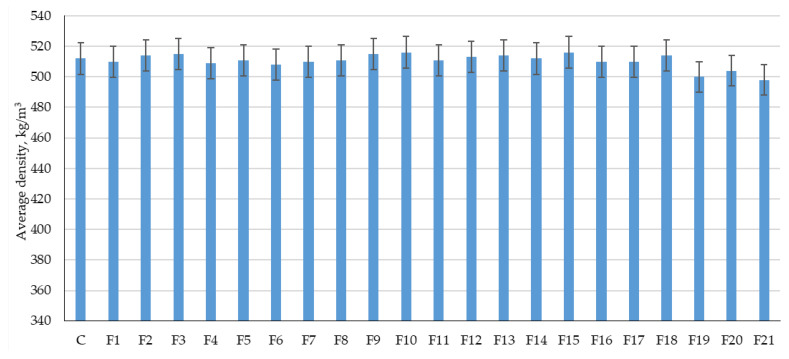
Results of experimental studies of the average density of fiber foam concrete samples at various dosages of microsilica introduced instead of a part of the binder and dosages of polypropylene fiber of 0%, 1%, 2%, 3% (designation, see Table 5).

**Figure 12:**
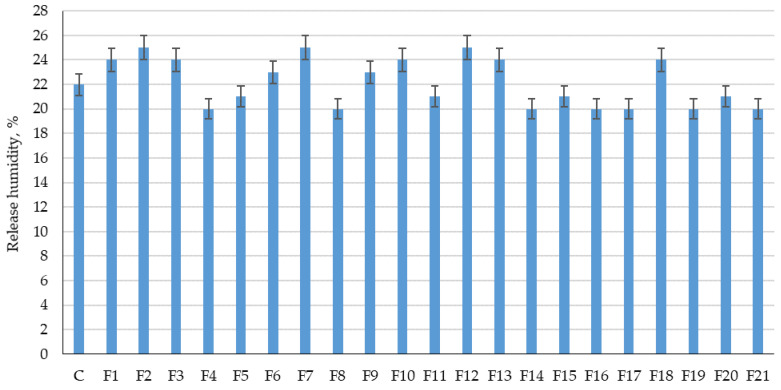
Results of experimental studies of the release moisture content of fiber foam concrete samples at various dosages of microsilica introduced instead of a part of the binder and dosages of polypropylene fiber of 0%, 1%, 2%, 3%.

The results of experimental studies of the influence of the content of microsilica additive introduced instead of a part of cement, and the percentage of dispersed reinforcement with polypropylene fiber on the physical characteristics of fiber foam concrete are presented in Figure 11 and Figure 12.

Figure 11 and Figure 12 show the results of determining the average density and release moisture content of the test samples of fiber foam concrete.

From Figure 11 and Figure 12, it follows that those different dosages of microsilica introduced instead of a part of the binder and the different dosages of polypropylene fiber do not have any significant effect on the change in average density and release moisture. The average density varied from 500 to 516 kg/m^3^, and the release moisture values ranged from 20% to 25%. Thus, the analysis of Figure 11 and Figure 12 did not reveal a clear dependence of density and release moisture on the dosage of fiber and nanomodifier.

**Figure 13:**
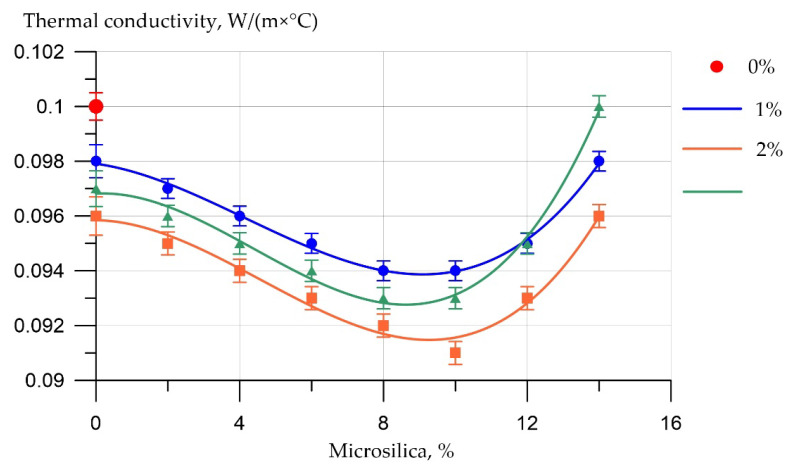
Results of experimental studies of the thermal conductivity of fiber foam concrete samples at various dosages of microsilica introduced instead of a part of the binder and dosages of polypropylene fiber of 0%, 1%, 2%, 3%.

Figure 13 shows the results of determining the thermal conductivity of test samples of fiber foam concrete.

The best value of the thermal conductivity of the test samples of fiber foam concrete was recorded for the composition of type F14, where the replacement of part of the cement with the addition of silica fume was 10%. Thus, as in the case of strength characteristics, the dosage of microsilica of 10% is the best. As for the percentage of fiber reinforcement, at a dosage of 2%, the lowest values of thermal conductivity are observed.

### 3.4. Study of the Structure of Fiber Foam Concrete with Polypropylene Fiber and Microsilica

**Figure 14:**
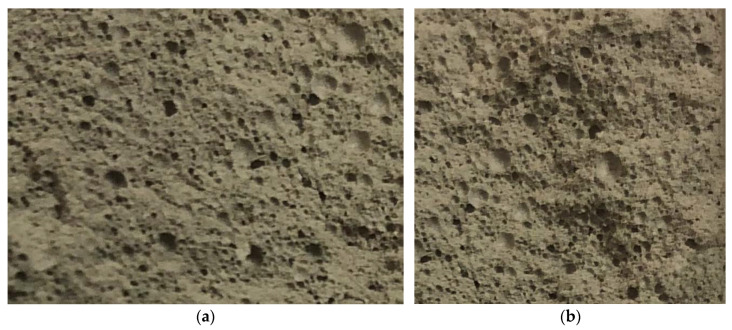
Pore macrostructure (with a magnification of 5 times): (**a**) control sample; (**b**) a sample of fiber-foam concrete using nanomodified microsilica and dispersion reinforced with polypropylene fiber.

Figure 14a shows the pore macrostructure of the control sample. Figure 14b shows the pore macrostructure of a fiber foam concrete sample using nanomodified silica fume and dispersed/reinforced with polypropylene fiber.

The photographs show differences in the pore macrostructure between the two samples. So, the porous macrostructure of the control sample of foam concrete in Figure 14a turned out to have unreasonably thickened interpore partitions, which in turn are characterized by microporosity, and, thereby, the strength characteristics of such foam concrete are reduced. At the same time, macropores are insufficiently monofractional, are chaotic in nature and, due to their small size, ultimately adversely affect the thermal insulation properties of such foam concrete. At the same time, Figure 14b shows a sample of foam concrete based on silica fume dispersed/reinforced with polypropylene fiber. Here, a significant enlargement of the main pores is already noticeable, due to a decrease in the microporosity of the interpore partitions, a relatively high monodispersity of the pores is observed, which has a positive effect on the thermal insulation properties of the resulting concrete.

**Figure 15:**
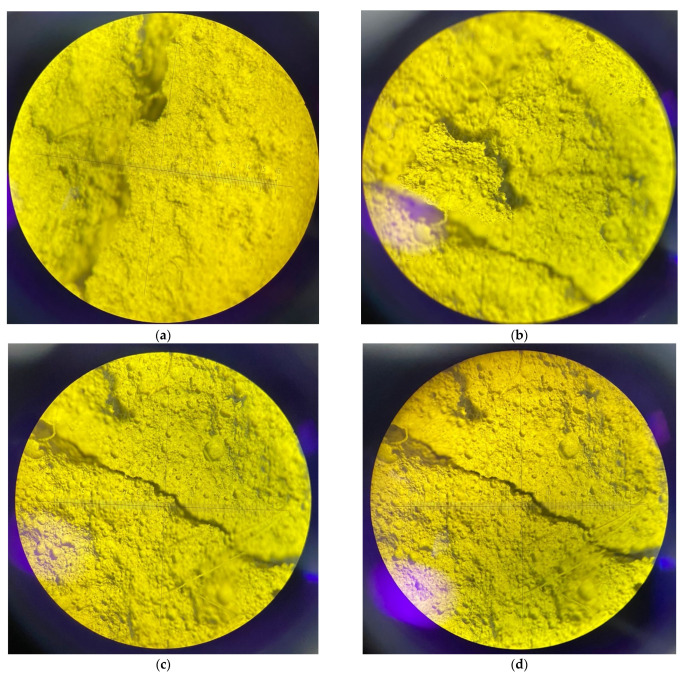
Photographs of the microstructure of the obtained sample of fiber foam concrete nanomodified with microsilica with a magnification of 6 times: (**a**) the nature of the destruction of concrete and the exposure of reinforcing fibers at the fracture of the sample; (**b**) the nature of the displacement of structural fragments in the deformation zones during ductile fracture; (**c**) the nature of the crack at the initial stage of failure with a small crack opening; (**d**) demonstration of the stability of the concrete structure to maintain the width of the crack opening with increasing load (performed on an MBS-10 microscope).

Next, consider Figure 15, which shows the microstructure of the obtained sample of fiber foam concrete nanomodified with microsilica.

After testing on the specified sample, the absence of microcracking at the phase boundary between the polypropylene fiber and the reinforced cement–sand matrix due to nanomodification is well monitored. The nature of the micropore structure makes it possible to register the positive influence of the combined effect of dispersed reinforcement and nanomodification. Thus, on the example of the fundamental relationship between the composition of the micro- and macrostructure and the properties of the material, the positive influence of the proposed recipe–technological factors has been proved.

Let us analyze the mechanism of the processes occurring in the fundamental nature of the structure formation of foam concrete of the obtained type. Due to the fact that at the micro level the pore structure acquires a denser packing of particles, microporosity decreases, macroporosity increases due to the strengthening of interpore partitions, volume is preserved, and concrete shrinkage is reduced. Thus, the density of the material, due to the preservation of the volume at the same level, is reduced. At the same time, interpore partitions acquire a denser packing of particles at the micro level, and at the macro level the foam concrete structure acquires a more perfect pore structure, the pores become monodisperse, large, and all this has a positive effect on the thermal insulation properties of the final product [43].

When fiber is added, the internal forces in the concrete are redistributed. Those microcracks that occurred in the absence of fiber reinforcement under mechanical, static, and dynamic effects on such concrete are absent with the introduction of fiber, which has some damping, softening effect. The reduction of microcrack formation, which has been established by studies both at the macro level and at the micro level with the help of a microscope, makes it possible to improve not only the structure of this concrete but also its properties [44,45,46].

The combined effect of nanomodification with microsilica and fiber reinforcement with polypropylene fibers improves both the mechanical and physical properties of foam concrete. It is in this connection that its strength indicators, as well as the thermal conductivity index, have improved.

It is known that the strength of cellular concrete largely depends on the physical and mechanical properties of the material of interpore partitions, in particular, on its density. However, in real materials, dense packing of solid particles in interpore walls is usually not achieved due to the angularity and roughness of the particles [3,29,35,47,48]. In the present study, the effective compaction of the material of interpore partitions to eliminate capillary porosity in them and increase the area of phase contacts between particles was achieved using a nanomodifying microsilica additive. Microsilica acts as a filler, it provides a denser packing of particles, and also promotes the additional formation of calcium silicate hydrate (CSH) [36,49,50,51]. Thus, a larger amount of CSH contributes to a denser packing of particles of inert components in the material of the interpore wall as well as faster curing of foam concrete and, accordingly, higher values of compressive strength [30,52,53].

Let us carry out a comparative analysis of the results obtained in this study with data obtained by other authors. In [19], the effect of various dosages from 0.25% to 0.55% of alkali-treated banana fiber on the thermal conductivity coefficient of ultralight fiber foam concretes was studied, where, according to the research results, the best value of the thermal conductivity coefficient was recorded at a fiber dosage of 0.55%, and its value itself decreased by 13%. In our case, the lowest value of the thermal conductivity coefficient was recorded at the content of polypropylene fiber in the amount of 2% by weight of the binder. At a given dosage of fiber, its value decreased by only 4% for foam concrete reinforced with fiber and by 9% for fiber foam concrete, additionally modified with the addition of microsilica, compared with the control value of a sample not reinforced with fiber. Thus, it can be concluded that the use of polypropylene fiber in comparison with banana fiber has a lesser effect on the thermal conductivity of this material.

In [54], the effect of recycled PET fiber on the mechanical characteristics of foam concrete was studied. This fiber was considered as a dispersed reinforcing additive. Based on the results of the study, the authors determined the optimal dosage of the fiber and its length, which amounted to 0.1% and 6 mm, respectively. Thus, at a given percentage of reinforcement with recycled PET fiber, the increase in compressive strength was 11.5% compared to the control composition. In the present study, the use of polypropylene fiber in an amount of 2% by weight of the binder provides an increase in compressive strength up to 18%, and in combination with a nanomodifier, the value of this increase is about 44%. From this it should be emphasized that the use of recycled PET fiber in comparison with polypropylene fiber does not provide such a high increase in strength characteristics.

In [30], the authors studied the effect of microsilica and basalt fiber on the mechanical characteristics of foam concrete. According to the results of experimental studies, it was found that the addition of 15% microsilica increased the compressive strength up to 46%, while increasing the content of basalt fiber from 0 to 3% increased the bending strength up to 88%. In the present study, at the best dosages of fiber (2%) and microsilica (10%), the maximum increases in compressive strength and tensile strength in bending were 44% and 73%. In general, the compositions “microsilica–basalt fiber” and “microsilica–polypropylene fiber” are effective and provide a fairly high increase in the strength characteristics of foam concrete.

Comparison with works [2,29,31] confirmed the effectiveness of prescription methods given in this study, the results of which are consistent with the results obtained by the authors in [2,29,31].

## 4. Conclusions

Thus, based on the results of the study, the following conclusions can be drawn:

(1) The use of polypropylene fibers in the range from 1% to 3% by weight of the binder makes it possible to improve the mechanical and physical characteristics of fiber foam concrete; the best values of the strength and physical characteristics of fiber foam concrete are recorded at a dosage of polypropylene fiber of 2%;
(2) The optimal dosage of the nanomodifying microsilica additive introduced instead of a part of the binder is 10%;
(3) The maximum values of increments in mechanical characteristics were 44% for compressive strength and 73% for tensile strength in bending, the values of the coefficient of thermal conductivity of fiber foam concrete at optimal dosages of the nanomodifier and polypropylene fiber decreased by 9%, and the use of polypropylene fiber and microsilica in the ranges considered in this study does not reflect a clear dependence of density and release moisture on the dosage of fiber and nanomodifier;
(4) The results obtained in the course of the study make it possible to determine further research prospects for structural, heat-insulating, and structural foam concrete and considering the possibility of improving their characteristics and significantly increasing the reliability of enclosing load-bearing structures made of such materials.

## Figures and Tables

**Table 1:** Comparison of foam concrete and aerated concrete.

Foam Concrete	Aerated Concrete
Main raw materials	
Cement, sand, water and foam, microsilica, fly ash, ground blast furnace granulated slag [8]	lime, cement, sand, water and aluminum powder, microsilica, fly ash, ground blast furnace granulated slag [9]
Technology distinguishing equipment	
Foam generator [6,8,10]	Autoclave plant [1,9]
Environmental impact	
No pollution (low energy consumption) [6,8]	High energy consumption [1,9,11,12,13]

**Table 2:** Overview of various methods for modifying and improving the physical and mechanical characteristics of foam concrete and foamed geopolymers.

Ref.	Improving Method	Primary Results
[14]	Modification of foam concrete with magnetite (Fe_3_O_4_) nanoparticles	The use of magnetite nanoparticles in an amount of 0.25% by weight of the binder gives the best characteristics in terms of compressive strength, bending, and tensile splitting; also the use of Fe_3_O_4_ increases the viscosity and yield strength of the foam concrete mixture.
[15]	The use of cow hair as a dispersed reinforcing fiber	The optimal content of cow hair in foam concrete is the dosage in the amount of 0.2% by weight. This foam concrete has the best mechanical properties: the increase in compressive strength was 68.37%, and the bending strength increased by 50%.
[16]	Application of dolomite powder	The use of dolomite powder contributes to the accelerated curing of foam concrete.
[17]	Application of fly ash additive	The use of fly ash additive slightly increases the density of foam concrete and reduces shrinkage; however, the low rate of fly ash hydration adversely affects the compressive strength of foam concrete.
[18]	Modification of foam concrete with nanotubes	The use of nanotubes in foam concrete increases the compressive and tensile strength and provides more efficient growth of the addition of grains C–S–H.
[19]	Applications of alkali-treated banana fiber	It has been established that when banana fiber is used in foam concrete with a dosage of 0.1% to 0.6%, water absorption improves as the dosage increases, while concrete porosity, on the contrary, decreases.
[20]	Application of dispersed reinforcing coconut fiber	Samples of foam concrete with the addition of coconut fiber in an amount of 0.35% showed the best compressive strength values.
[21]	Applications of nanoparticles	It has been established that the optimal content of nanoparticles makes it possible to improve the mechanical properties of freshly prepared foam concrete as well as some physical characteristics, such as thermal conductivity, water absorption, and shrinkage.
[22]	The use of fibers from the stem of the oil palm tree	The use of fibers from the trunk of an oil palm tree in foam concrete contributes to a significant improvement in strength characteristics and reduces shrinkage during drying.
[23]	Use of quarry dust waste	The use of quarry dust as a replacement for fine aggregates from 75% to 100% contributes to an increase in strength characteristics and reduces the fluidity of foam concrete mixtures.
[24]	Application of styrofoam particles	The use of expanded polystyrene particles in foam concrete provides a significant reduction in the thermal conductivity and fire resistance of the material and also reduces the strength characteristics.
[25]	Application of fly ash and microsilica	According to the results of the study, the authors found that the use of microsilica is more effective than fly ash; samples with a high foam content and the addition of microsilica showed the best values for compressive strength and thermal conductivity.
[26]	Application of hydrophobic starch nanoparticles	Foam made using starch nanoparticles has increased stability and improved density and viscosity. The compressive strength increased from 2.35 MPa to 2.77 MPa. The percentage of pores in the range of 100–500 µm increased to 63.2%, which means that the addition of starch nanoparticles makes the pore size distribution narrower, and the pore size is smaller.
[27]	Application of sisal fibers	When the content of sisal fiber is less than 0.15%, the higher the content of sisal fiber, the greater the bending strength and fatigue life of foam concrete. If the sisal fiber content is greater than 0.15%, the flexural strength and fatigue life decrease as the fiber content increases. The optimal content of sisal fiber in foam concrete is 0.133%
[28]	The use of natural fibers	The fiber foam concrete was reinforced with henequen natural fibers (raw or alkali treated) in volume fractions of 0.5%, 1%, and 1.5%. Polypropylene fiber reinforcement was also used as a reference. For all fiber-reinforced concretes, the inclusion of fibers increased the compressive and tensile strength and plastic behavior, which was associated with an increase in the integrity of the sample. Microscopic characterization showed that the inclusion of fibers did not change the size of the air cavity and its distribution.
[2,5,29,30,31,32,33]	The use of polypropylene, basalt and fiberglass	Improves the mechanical and plastic properties of foam concrete, contributes to better tensile performance of the composite

**Table 3:** Characteristics of raw materials.

Material Name	Characteristics	Actual Value
Portland cement CEM I 42.5N (Novoroscement, Novorossiysk, Russia)	Specific surface, m^2^/kg	337
	Normal density of cement paste, %	26
	Grinding fineness, sieve pass No. 008%	98.7
	Setting time, min	
	- start - the end	160 235
	Tensile strength in bending, MPa:	
	- 2 days - 28 days	4.3 7.8
	Compressive strength, MPa:	
	- 2 days - 28 days	25.1 46.8
Quartz sand (Arkhipovsky quarry, v. Arkhipovskoe, Russia)	Size modulus	0.82
	Bulk density, kg/m^3^	1423
	True density, kg/m^3^	2878
	Content of dust and clay particles, %	0.5
	Clay content in lumps, %	0.1
	Content of organic and contaminants	absent
Foam generating agent PB-2000 (Ivkhimprom, Ivanovo, Russia)	Density, kg/m^3^	1200
	Hydrogen index (pH) of the blowing agent	8
	Foam stability, s	360
Polypropylene fiber (Fibropolymer, Moscow, Russia)	Fiber length, mm	6
	Diameter, µm	20–25
	Density, kg/m^3^	910
	Strength, MPa	570
	Lubricant	Silastol CUT 70
	Humidity, %	2
	Absorption	No

**Table 4:** Chemical composition of microsilica.

Title	Actual Content, %
Na_2_O	1.022
MgO	1.563
Al_2_O_3_	0.453
SiO_2_	90.987
P_2_O_5_	0.125
SO_3_	0.696
Cl	0.220
K_2_O	1.971
CaO	0.522
Cr_2_O_3_	0.002
MnO	0.123
Fe_2_O_3_	2.261
NiO	0.010
CuO	0.010
ZnO	0.035
TiO_2_	–

**Table 5:** Experimental research program.

Composition Number	MS, %	Fiber, %	Type, Size and Number of Samples				
			Compressive Strength	Average Density	Release Humidity	Flexural Tensile Strength	Thermal Conductivity
C	0	0	Cubes 100 × 100 × 100 (mm) (3 × 22 = 66 pcs)			Prisms 100 × 100 × 400 (mm) (3 × 22 = 66 pcs)	Plates 100 × 100 × 20 (mm) (3 × 22 = 66 pcs)
F1	2	1					
F2	2	2					
F3	2	3					
F4	4	1					
F5	4	2					
F6	4	3					
F7	6	1					
F8	6	2					
F9	6	3					
F10	8	1					
F11	8	2					
F12	8	3					
F13	10	1					
F14	10	2					
F15	10	3					
F16	12	1					
F17	12	2					
F18	12	3					
F19	14	1					
F20	14	2					
F21	14	3					

## Data Availability

The data presented in this study are available on request from the corresponding author.
